# Association of intraoperative pulse pressure drop and minimum mean arterial pressure with postoperative length of stay: A stratified reanalysis of published data by age and sex

**DOI:** 10.1371/journal.pone.0350048

**Published:** 2026-05-28

**Authors:** Victor Beaucoté, Jérôme Cartailler, Jérémie Mattern, Bernard Trillat, Etienne Gayat, Morgan Le Guen, Marc Fischler

**Affiliations:** 1 Department of Anesthesiology and Critical Care Medicine, Hôpital Lariboisière, Paris, France; 2 U942 MASCOT, Inserm, Université Paris Cité, Paris, France; 3 Department of Obstetrics and Gynecology, Hôpital Antoine Béclère, Clamart, France; 4 Department of Information Systems, Hôpital Foch, Suresnes, France; 5 Department of Anesthesiology, Hôpital Foch, Suresnes, France; Scuola Superiore Sant'Anna, ITALY

## Abstract

**Objective:**

Intraoperative hypotension (IOH) is associated with postoperative organ dysfunction, but no universal definition exists. Guidelines recommend a mean arterial pressure (MAP) threshold of 60–70 mmHg. Prolonged postoperative length of stay (pPOLOS) serves as a proxy for organ injury. This study explores the association between IOH and pPOLOS risk, accounting for patient characteristics (sex and age).

**Design:**

Retrospective, single-center cohort study of adult patients undergoing general anesthesia for scheduled non-cardiac surgery between July 2017 and December 2019.

**Methods:**

pPOLOS was defined as a LOS higher than the median value (main outcome). Relationships between pPOLOS risk and three previously identified IOH variables—drop in pulse pressure (DropPP: difference between maximum and minimum values), minimal MAP (MinMAP), and cumulative time with pulse pressure > 61 mmHg per hour of surgery (CumTimePP > 61 mmHg)—were modeled using piecewise linear splines.

**Results:**

Our study examined 9,516 patients. For the whole population, the relationship between DropPP and pPOLOS risk was pseudolinear with no activation threshold (slope 0.29–0.52%/mmHg). For MinMAP, an activation threshold of 73 mmHg was identified, below which the association became linear (slope: −0.64%/mmHg). For CumTimePP > 61 mmHg, pPOLOS risk increased sharply (initial slope: 8.40%/min) and reached a saturation threshold at two minutes. Women demonstrated a lower pPOLOS risk and a lower IOH threshold than men. In contrast, older patients (≥65 years) exhibited a higher baseline pPOLOS risk and showed no identifiable IOH threshold.

**Conclusions:**

The identified IOH threshold (MAP < 73 mmHg) is in line with values reported in the existing literature. Pulse pressure variability, age, and sex emerged as key determinants of pPOLOS risk.

## Introduction

Intraoperative hypotension (IOH) is a common event during general anesthesia with multifactorial etiologies. IOH is associated with postoperative morbidity: acute kidney injury [1], myocardial ischemia [2], and mortality [3]. However, routine screening for organ damage is not systematically performed on every patient as this requires close clinical or biological monitoring practice used only in high-risk populations. Consequently, such studies may underestimate IOH harm.

Postoperative length of stay (POLOS) represents an objective, readily ascertainable outcome available for all surgical patients. While POLOS is strongly influenced by severe postoperative complications, it is also substantially affected by non-clinical factors, particularly social determinants [4], making it a potential surrogate endpoint for cumulative organ injury as demonstrated in a cohort of severely injured patients [5]. Currently, the relationship between IOH and POLOS remains understudied and has primarily been examined as a secondary outcome [6]. Further investigation is required to elucidate the magnitude and clinical relevance of this association.

Current consensus guidelines recommend maintaining an intraoperative mean arterial pressure (MAP) > 60–70 mmHg, regardless of patient characteristics [7]. This unified recommendation avoids complex, component-specific targets depending on the component of arterial pressure (systolic, diastolic, mean, and pulse) and its value (minimal value, area under the curve...). Similarly, this recommendation does not take into consideration the patient's baseline blood pressure (usual or that measured immediately preoperatively).

Advances in data analyses can help the choice of the most relevant IOH component for organ injury evaluation. Using an unsupervised clustering method, our team showed in a previous study that three intraoperative IOH variables are associated with prolonged POLOS (pPOLOS) when POLOS is defined as > 50^th^ percentile: drop in pulse pressure (DropPP:difference between maximum and minimum intraoperative pulse pressure–PP–values), minimal value of mean arterial pressure (MinMAP), and CumTimePP > 61 mmHg (cumulative time of PP spent over 61 mmHg per hour of surgery) [8]. We also reported that DropPP and MinMAP are associated with pPOLOS (> 75th percentile), whereas DropPP is the sole associated variable when the threshold is increased to the > 90th percentile.

The aim of the present study was to characterize the relationship between pPOLOS risk (defined as LOS > 50^th^ percentile within surgery type and duration subgroups) and each of these IOH variables, stratified by age and sex. Secondary outcomes examined pPOLOS defined as > 75^th^ and > 90^th^ percentiles.

## Methods

### Study design, ethics approval and setting

This retrospective single-center study was conducted at a private, non-profit tertiary academic hospital that performs approximately 20,000 anesthetic procedures annually across diverse surgical specialties, excluding cardiac surgery. The study was approved by the local Ethics Committee (Chairperson: Professor Hervé) on December 18, 2019 (n° 19-11-3). Patients were informed via hospital notices that their anonymized data could be used for research purposes, along with clear instructions on how to opt out of the study. This information included the necessary information to enable them to refuse their participation. As a result of this procedure, the need for consent was waived by the Ethics Committee. Data were accessed for research purpose from 28/01/2020 to 25/02/2020. Authors had no access to information that could identify individual participants during or after data collection. This report follows the Strengthening the Reporting of Observational Studies in Epidemiology (STROBE) guidelines for observational cohort studies.

### Patient population

The analysis included all patients aged 18 years or older who underwent general anesthesia between July 15, 2017 and December 31, 2019 and stayed in hospital for at least one night. Patients were excluded if operating time was less than 20 min, if they had anesthesia more than once during the same hospitalization, and if they had an obstetric surgical procedure, lung transplantation, interventional radiology, and gastrointestinal endoscopy and bronchoscopy. Patients with no recorded arterial pressure signal or with aberrant or incomplete signal values were also excluded. All patients were managed according to usual recommendations, especially regarding intraoperative monitoring.

### Data collection

The details of data collection and IOH variables construction and selection have been previously published [8].

### Outcomes

The primary outcome was pPOLOS. First, POLOS was calculated as the number of days between the surgical intervention and hospital discharge. Then, patients were grouped by surgery specialty: digestive, thoracic, gynecological, neurosurgery, otorhinolaryngology, urology, and vascular procedures (7 classes). Within each surgery group, patients were divided into four sub-categories based on surgery duration quartiles, which gave 28 sub-groups in total. Within each subgroup, patients were classified as having pPOLOS if their LOS exceeded the 50^th^ percentile of that subgroup. The main objective concerned pPOLOS defined as > 50^th^ percentile.

The first secondary outcome concerned pPOLOS defined as > 75^th^ percentile conditioned to have an interquartile range (IQR) > 2 days (to break ties), otherwise we labelled patients using the modified formula pPOLOS > median + 1 day. The second secondary outcome concerned pPOLOS defined as > 90^th^ percentile. Finally, patients deceased after the surgery during hospitalization were considered in each pPOLOS category.

### Statistical analyses

To calculate the sample size, we set a significance level of 0.05 and a power of 80% (beta = 0.2). Using Hsieh's method [9], we anticipated a binary outcome (pPOLOS) with an odds ratio (OR) of 1.2 for the primary covariate. We assumed a non-informed scenario for prevalence with P0 set at 0.5. Given the robust hierarchical interaction among the blood pressure variables, we adjusted the sample size for multiple variables by applying a correction based on a high squared multiple correlation coefficient 0.95. This adjustment led to the required sample size of 4,740 patients.

Continuous variables were presented as median [IQR] or mean ± standard deviation as appropriate and absolute (n) or relative (%) frequencies in categorical variables. Statistical significance was defined as a *p*-value < 0.05. We applied the χ^2^ test and Student's t-test (or Mann-Whitney test if variables have non-normal distribution) to assess the differences between categorical and continuous variables. For descriptive analyses, missing values assumed to be missing completely at random (MCAR) were excluded at the variable level rather than at the patient level. Accordingly, no imputation method was applied.

To model the association between PLOS risk and each BP variable, we fitted piecewise linear continuous splines with 0 (purely linear), 1, or 2 knots (S1 Appendix, panel A). Model selection relied on the Bayesian Information Criterion (BIC), defined as BIC = −2 log L + p ln(n), where L is the likelihood, p is the number of estimated parameters, and n is the sample size, thereby balancing goodness of fit and model complexity. For the 1-knot and 2-knot candidates, knot locations were chosen by an exhaustive grid search over the predictor scale: the search domain was centered on the population median and bounded by ±2 standard deviations, with a resolution of 1 unit (e.g., 1 mmHg for MinMAP and DropPP). For 2-knot models, all ordered pairs in this grid were evaluated. For each variable (DropPP, MinMAP, and CumTimePP > 61 mmHg), the final model (0, 1, or 2 knots) was the candidate with the lowest BIC. After selecting the model, we characterized the fitted pattern as pseudo-linear, activation, or saturation based on the change in slope between adjacent segments at the knot. Specifically, pseudo-linearity was defined when the absolute slope change lay between −0.5 and +0.5% per mmHg (or % per min); slope increases greater than +0.5 were classified as activation, whereas decreases less than −0.5 were classified as saturation (S1 Appendix, panel B). This procedure was applied to the full cohort and to subgroups (women, men, age < 65 years, and age ≥ 65 years).

## Results

**Fig 1:**
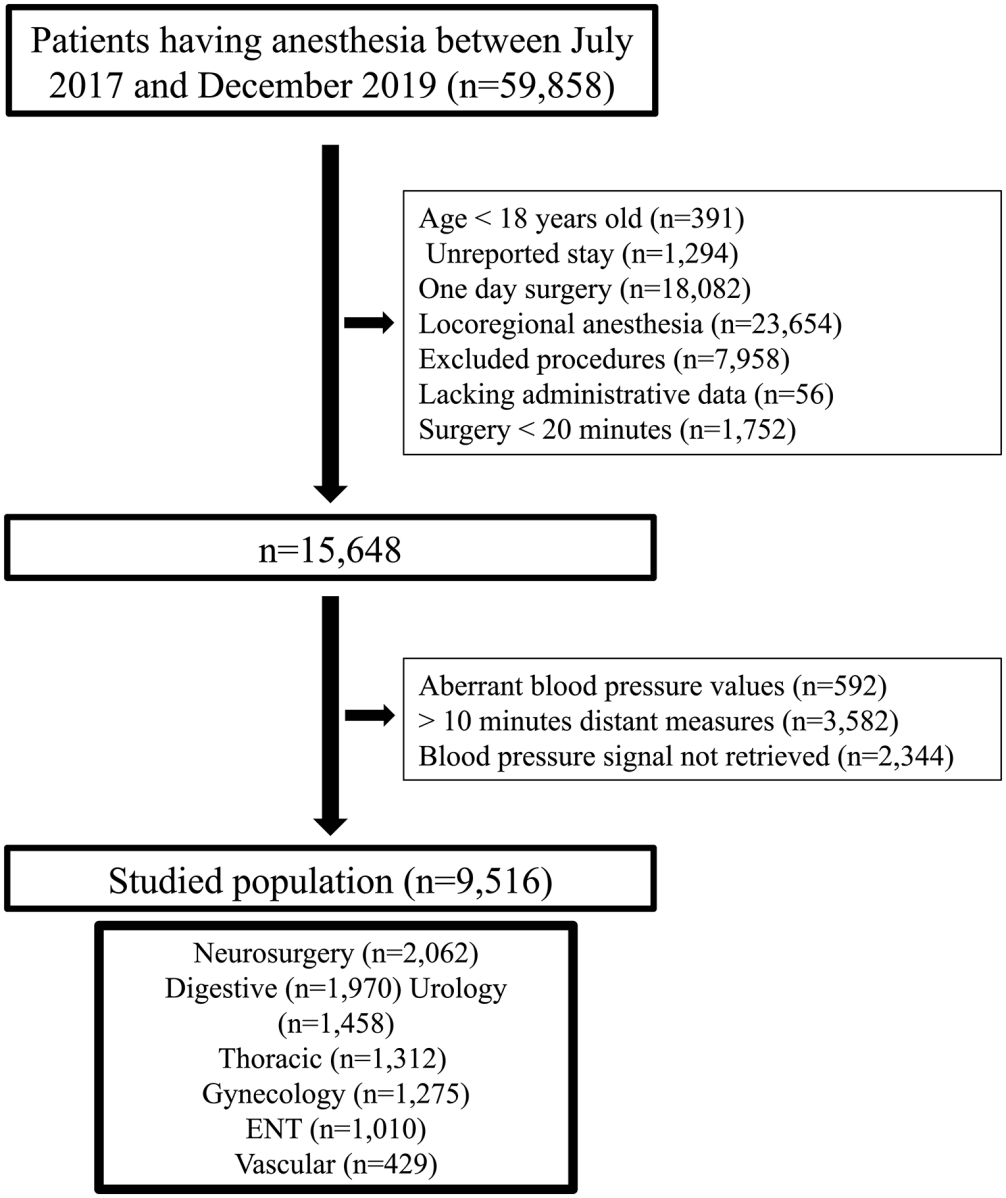
Flow chart.

A total of 59,858 patients were potentially eligible for inclusion. After exclusion, 9,516 patients were recruited for the study (**Fig 1**).

A total of 3,227 patients (33.9%) experienced pPOLOS defined by the median threshold (pPOLOS^50^), 1,657 (17.4%) patients when pPOLOS is > 75^th^ percentile (pPOLOS^75^) and 832 (8.7%) patients when pPOLOS is > 90^th^ percentile (pPOLOS^90^). Two hundred and thirty patients (2.4%) died during hospitalization. Mean patient age was 56.3 ± 17.6 years, and 52.4% were women. History of hypertension was present in 20.3% of the patients. A radial arterial catheter with continuous BP measurement was used in 19.9% of the patients. Baseline characteristics and simple frequencies and proportions of the main outcomes, stratified by the exposures of interest, have been previously published [8].

### Prolonged pPOLOS defined as LOS > 50^th^ Percentile (main outcome)

The characteristics of relationship between DropPP, MinMAP, and CumTimePP > 61 mmHg and pPOLOS are reported in Table 1.

**Table 1:** Characteristics of relationships between IOH variables and pPOLOS risk when pPOLOS is defined as > 50^th^ percentile.

Variables	Groups	Knot	Spline shape	Slope n°1 (%/mmHg or %/min)	Slope n°2 (%/mmHg or %/min)
**DropPP**					
	All	47 mmHg	Pseudolinear	0.52	0.28
	Women	49 mmHg	Pseudolinear	0.45	0.36
	Men	47 mmHg	Pseudolinear	0.58	0.19
	< 65 years	47 mmHg	Pseudolinear	0.35	0.20
	≥ 65 years	45 mmHg	Pseudolinear	0.46	0.18
**MinMAP**					
	All	73 mmHg	Activation	−0.64	−0.01
	Women	70 mmHg	Activation	−0.61	−0.03
	Men	75 mmHg	Activation	−0.68	−0.04
	< 65 years	74 mmHg	Activation	−0.57	0.12
	≥ 65 years	80 mmHg	Pseudolinear	−0.27	−0.38
**CumTimePP > 61 mmHg**					
	All	2 min	Saturation	8.40	0.23
	Women	2 min	Saturation	9.20	0.21
	Men	21 min	Saturation	0.68	−0.04
	< 65 years	2 min	Saturation	5.89	0.03
	≥ 65 years	21 min	Saturation	0.56	−0.06

MinMAP: minimal mean arterial pressure over intervention

DropPP: difference between the larger and smaller pulse pressure values computed over the entire intervention

CumTimePP: cumulative time pulse pressure spent above 61 mmHg

pPOLOS: postoperative length of stay

Knot: intersection point of two linear splines

Pseudolinear: relationship defined by an absolute slope difference between two splines ranging in [−0.5; + 0.5] %/mmHg (or %/min)

Activation: relationship defined by an absolute slope difference between two splines > 0.5%/mmHg (or %/min)

Saturation: relationship defined by an absolute slope difference between two splines < −0.5%/mmHg (or %/min)

#### Prolonged pPOLOS^50^ risk and DropPP.

**Fig 2:**
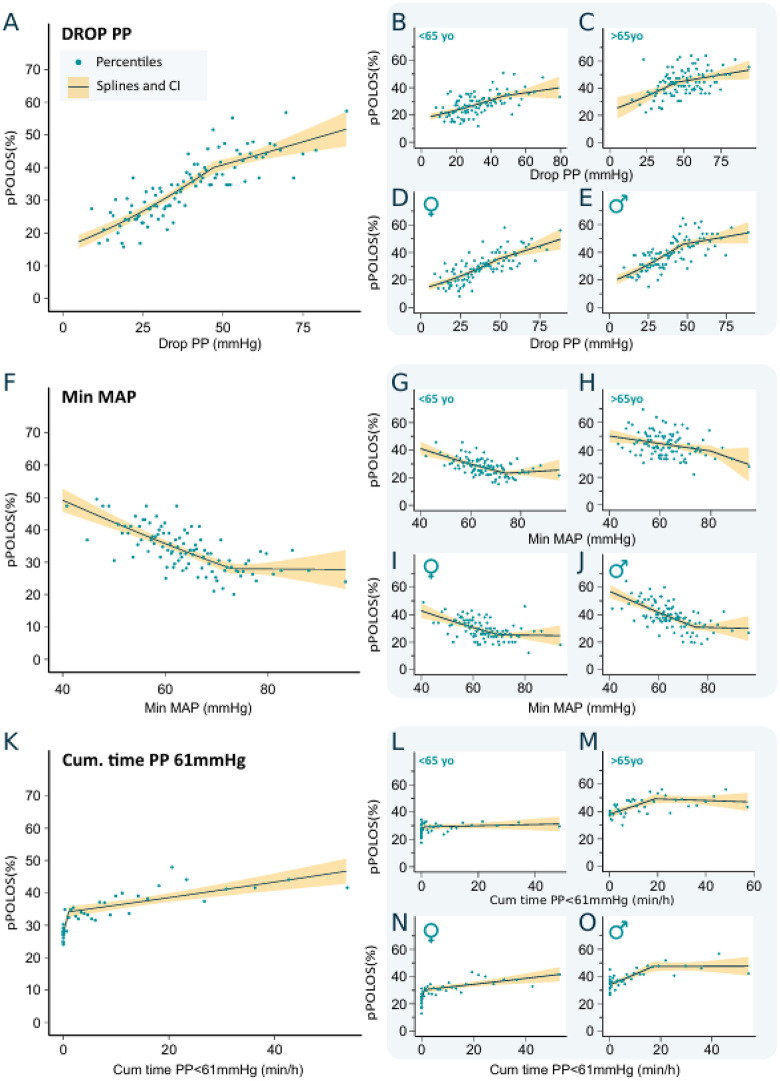
Relationship between IOH and risk of prolonged length of stay > 50th percentile (pPOLOS^50^). **DropPP:** Overall population (A), age < 65 and ≥65 years (B, C), and sex (women and men) (D, E). **MinMAP:** Overall population (F), age < 65 and ≥65 years (G, H), and sex (women and men) (I, J). **CumTimePP > 61 mmHg:** Overall population (K), age < 65 and ≥65 years (L, M), and sex (women and men) (N, O). DropPP: difference between the largest and smallest pulse pressure values over the entire intervention. MinMAP: minimal mean arterial pressure during anesthesia. CumTimePP > 61 mmHg: cumulative time pulse pressure spent above 61 mmHg. Solid lines represent splines from a generalized linear regression model, with shaded areas indicating 95% confidence intervals.

For the entire cohort, a pseudolinear increasing relationship was observed between DropPP and pPOLOS^50^ risk, with a significant risk increase between 10 and 47 mmHg at a rate of approximately 0.5% per mmHg (Fig 2, Panel A). The slope of risk increase ranged from +0.29 to +0.52%/mmHg.

Mean pPOLOS50 risk was lower in younger patients (< 65 years: 27.9 ± 44.8%) compared with older patients (≥ 65 years: 43.9 ± 49.6%). There was a positive relationship that was less clear among older patients (> 65 years) (**Fig 2, Panels B and C**). Men had higher mean pPOLOS^50^ risk compared with women (38.9 ± 48.8% vs 29.4 ± 45.5%). The slope of this relationship was not different among men and women (**Fig 2, Panels D and E**).

#### Prolonged pPOLOS^50^ risk and MinMAP.

For the entire cohort, the MinMAP activation threshold below which pPOLOS^50^ risk began to increase was 73 mmHg, with a slope of −0.64%/mmHg below this threshold (**Fig 2, Panel F**).

In the younger population (< 65 years), the MinMAP threshold was 74 mmHg. The slope of risk increase was −0.57%/mmHg under the threshold in this population. In the older population (≥ 65 years) there was no threshold MinMAP value under which the pPOLOS^50^ started to rise, there was a monotonic decreasing relationship (**Fig 2, Panels G and H**).

In men, the MinMAP threshold was higher than in women (75 mmHg vs 70 mmHg, respectively), but the slope of risk increases was similar in men and women, −0.68%/mmHg against −0.61%/mmHg, respectively (**Fig 2, Panels I and J**).

#### Prolonged pPOLOS^50^ risk and CumTimePP > 61 mmHg.

For the entire cohort, a saturation threshold of 2 minutes was identified, above which the relationship between CumTimePP > 61 mmHg and pPOLOS^50^ risk became flatter (**Fig 2, Panel K**). The slope of risk increase was + 8.39%/min and +0.226%/min for the < 2 minutes and ≥ 2 minutes portions, respectively. The threshold value was lower in younger patients (< 65 years) than in older ones with 2 minutes against 21 minutes **(Fig 2, Panels L and M**). The threshold value was higher in men with 21 minutes, against 2 minutes in women (**Fig 2, Panels N and O**).

### Prolonged pPOLOS defined as LOS > 75^th^ Percentile (secondary outcome)

The characteristics of relationship between DropPP, MinMAP, and CumTimePP > 61 mmHg and pPOLOS are reported in Table 2.

**Table 2:** Characteristics of relationships between IOH variables and pPOLOS risk when pPOLOS is defined as > 75^th^ percentile.

Variables	Groups	Knot (mmHg)	Spline shape	Slope n°1 (%/mmHg)	Slope n°2 (%/mmHg)
**DropPP**					
	All	36	Pseudolinear	0.62	0.13
	Women	49	Pseudolinear	0.43	0.09
	Men	35	Saturation	0.80	0.01
	< 65 years	36	Pseudolinear	0.48	0.15
	≥ 65 years	30	Saturation	0.97	−0.01
**MinMAP**					
	All	72	Activation	−0.79	−0.18
	Women	70	Activation	−0.90	−0.04
	Men	60	Pseudolinear	−0.29	−0.35
	< 65 years	74	Activation	−0.82	−0.03
	≥ 65 years	74	Pseudolinear	−0.40	−0.32

MinMAP: minimal mean arterial pressure over intervention

DropPP: difference between the larger and smaller pulse pressure values computed over the entire intervention; pPOLOS: postoperative length of stay

Knot: intersection point of two linear splines

Pseudolinear: relationship defined by an absolute slope difference between two splines ranging in [−0.5; + 0.5] %/mmHg (or %/min)

Activation: relationship defined by an absolute slope difference between two splines > 0.5%/mmHg (or %/min)

Saturation: relationship defined by an absolute slope difference between two splines < −0.5%/mmHg (or %/min)

#### Prolonged pPOLOS^75^ risk and DropPP.

**Fig 3:**
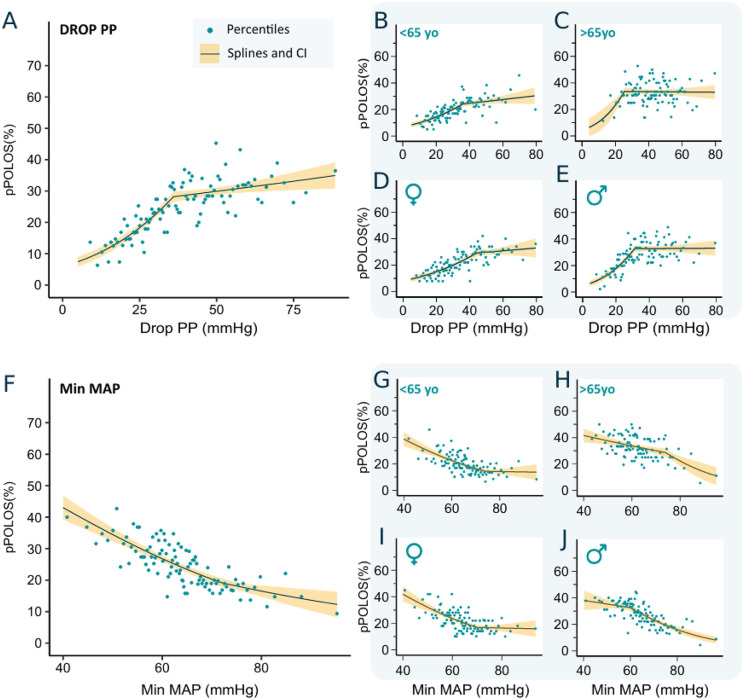
Relationship between IOH and risk of prolonged length of stay >75th percentile (pPOLOS^75^). **DropPP**: Overall population (A), age < 65 and ≥65 years (B, C), and sex (women and men) (D, E). **MinMAP**: Overall population (F), age < 65 and ≥65 years (G, H), and sex (women and men) (I, J). DropPP: difference between the largest and smallest pulse pressure values; MinMAP, minimal mean arterial pressure. MinMAP: minimal mean arterial pressure. Solid lines represent splines from a generalized linear regression model, with shaded areas indicating 95% confidence intervals.

For the > 75^th^ percentile pPOLOS definition (pPOLOS^75^), the DropPP/pPOLOS relationship had similar characteristics as the > 50^th^ percentile one. In the whole cohort, there was a pseudolinear relationship between DropPP and pPOLOS^75^; for example, between 10–30 mmHg, the risk of pPOLOS sharply increases from 8% to 28% (**Fig 3, Panel A**). In both ≥ 65 years old and men subgroups, there was a saturation threshold (35 mmHg and 30 mmHg, respectively) (**Fig 3, Panels B to E**).

#### Prolonged pPOLOS^75^ risk and MinMAP.

The relationship between MinMAP and risk of pPOLOS^75^ also presents an activation threshold at 72 mmHg (**Fig 3, Panel F**). This trend was consistent across women and younger patients (< 65 years). In both men and ≥ 65 years old subgroups, there was a pseudolinear relationship without any activation threshold (**Fig 3, Panels G to J**).

### Prolonged pPOLOS defined as LOS > 90^th^ Percentile (secondary objective)

**Fig 4:**
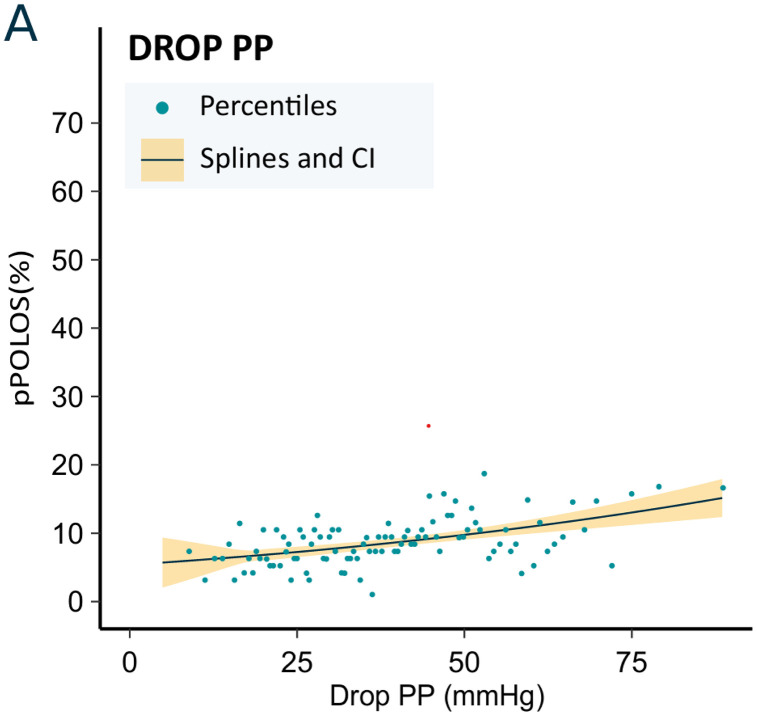
Relationship between DropPP and risk of prolonged length of stay >90^th^ percentile (pPOLOS^90^) in the entire population. DropPP: difference between the largest and smallest pulse pressure values.Solid lines represent splines from a generalized linear regression model, with shaded areas indicating 95% confidence intervals.

We finally investigated the relationship between DropPP and pPOLOS defined using only the length of stay longer than the 90^th^ percentile (pPOLOS^90^). As above, DropPP exhibits a pseudolinear relationship but with a lower slope (**Fig 4**). More details (knot, threshold, and slope) cannot be provided considering the small number of patients. Similarly, further description for age and sex subgroups is not provided because of the limited number of patients.

## Discussion

Our recently published study identified three variables statistically associated with pPOLOS^50^: DropPP, MinMAP, and CumTimePP > 61mmHg [8]. In the present analysis, we characterized the relationship between these variables and pPOLOS risk across different pPOLOS definitions, stratified by patient age and sex.

POLOS functions as a pragmatic surrogate for complications in large-scale analyses, capturing objective data that circumvent underreporting biases [10]. Nevertheless, its validity is compromised by multiple confounding factors: institutional discharge practices, social determinants, logistical constraints, and financial pressures [11,12]. Additionally, enhanced recovery after surgery protocols reduce length of stay while obscuring post-discharge morbidity, further dissociating PLOS from true surgical morbidity. Finally, this metric lacks clinical specificity regarding complication type and severity, prevents causal inference, and suffers from definitional heterogeneity across studies (> 50th percentile [13], > 75th percentile [11] and > 90th percentile [14]), thereby limiting comparability.

Pulse pressure represents the pulsatile component of the blood pressure curve. It arises from the interaction between cardiac ejection and the elastic properties of the arterial circulation [15] and its change is proportional to volume change but inversely proportional to arterial compliance. Consequently, several pathological conditions result in widened PP particularly: aortic arteriosclerosis, since stiffening of the aorta and large arteries reduced arterial compliance and alter wave reflection [15]. From this perspective, it should be noted that the average age of our patients is 55.0 ± 17.5 years and that 13.9% are classified as having high blood pressure [8]. On the other hand, narrow PP occurs in conditions characterized by reduced stroke volume or cardiac output [16]. PP should not be confused with arterial pulse pressure variation with mechanical ventilation [17]. We observed a pseudolinear association between higher DropPP values and increased pPOLOS risk. DropPP has not yet been previously investigated as an IOH variable. In our cohort, the observed deleterious association with high DropPP values may reflect two phenomena. First, patient frailty may act as a confounding factor. The heterogeneous distribution of DropPP values may partly account for our findings, as the most vulnerable patients tend to exhibit higher DropPP levels. A subgroup analysis of a young non-comorbid cohort would be interesting to explore this bias. The second is a IOH deleterious effect per se. A high drop of PP may suggest a drop in stroke volume with tissue perfusion impairment. We can assume that prolonged intra-operative hypoperfusion leads to post-operative complications and may increase pPOLOS.

Numerous minimal arterial pressure thresholds have been proposed for postoperative organ dysfunction, including acute kidney injury, myocardial ischemia, stroke, delirium, and mortality. A 2018 meta-analysis by Wesselink et al. examining IOH-induced organ dysfunction demonstrated that IOH thresholds range from 60 to 80 mmHg depending on the outcome studied and IOH duration [18]. In 2019, the Perioperative Quality Initiative proposed an IOH MAP threshold of 60–70 mmHg to reduce the risk of postoperative organ dysfunction [7]. In our study, we identified an association between intraoperative MinMAP and pPOLOS, with an activation threshold of 73 mmHg. This finding is in line with IOH threshold literature regarding organ complications [2,19] and supports the assumption that pPOLOS is a good surrogate for global patient harm.

In our study, a longer time spent above 61 mmHg of PP was associated with increased pPOLOS. The maximum risk of pPOLOS was reached quickly, mainly after the first two minute per hour of surgery. This pattern suggests a separation between patients with short versus prolonged exposure to elevated PP, which might correspond to non-hypertensive and hypertensive profiles, respectively. However, the hypothesis that CumTimePP > 61 mmHg reflects underlying hypertensive status remains speculative, as we did not directly adjust for or stratify by documented hypertension in this analysis, and should therefore be interpreted with caution and confirmed in future studies.

As discussed earlier, the IOH MAP threshold seems to be around 60–70 mmHg. Some authors have reported different IOH thresholds depending on patient characteristics. For example, a higher IOH threshold (MAP < 80 mmHg) has been reported for elderly hypertensive patients undergoing major abdominal surgery when considering the risk of acute kidney injury [6], and increased mortality which has been found in hypertensive patients for the same IOH level and duration [3]. In our study, we examined IOH variables within subsets categorized by sex and age. Women demonstrated a lower MinMAP threshold (70 mmHg vs 75 mmHg in men). This difference may be explained by the higher comorbidity burden in men (mean ASA score 2.03 vs 1.84) or the age difference (mean age 59.0 vs 53.7 years). Physiological sex differences may also contribute to these findings. At steady state, men have higher BP values than women despite similar pulse pressure but the increase of BP with age is steeper in women [20,21]. Furthermore, the hypertension threshold above which cardiovascular risk starts to rise is lower in women [22]. These data are consistent with our finding of lower IOH threshold in women, with a BP range leftward shift. In older patients (≥ 65 years), pPOLOS risk was higher; however, no clear MinMAP activation threshold was identified. Such results may be explained by the loss of autoregulation function in comorbid patients (hypertension [23], diabetes mellitus [24]). As for the sex subset, vulnerability remains a strong confounding factor. The study of the MinMAP /pPOLOS relationship in a young non-comorbid cohort may be relevant.

Screening for postoperative organ dysfunction is primarily performed in high-risk patients, which may lead to underestimation of complication incidence in the general surgical population. The advantage of pPOLOS as an outcome measure lies in its ease of collection and completeness across all patients. The relationship between IOH and pPOLOS has been infrequently investigated, predominantly as a secondary outcome in small cohorts. Thus, some studies have reported an association between IOH and longer hospital stay after major abdominal surgery [25,26]. Sessler et al. reported in a large cohort that intraoperative occurrence of a triple low (MAP < 65 mmHg, bispectral index < 40, minimum alveolar concentration < 0.8) is associated with a prolonged LOS [13]. There are many factors associated with pPOLOS: age, functional status [27], nutritional state, medical/surgical complications, implementation of early recovery after surgery [28], pain or morphine use [29], or social factors [30]. Other unknown parameters can also influence pPOLOS, particularly logistic ones. Every patient's injury can increase pPOLOS by raising the incidence of complications which makes pPOLOS a surrogate for global harm [11]. However, the association between pPOLOS and complication onset remains controversial [31]. Some large multicentric cohorts have reported a weak association between pPOLOS and postoperative complications [11,32]. In those multicentric cohorts practice style differences may contribute to pPOLOS variations rather than complication onset. Our single-center cohort should be less affected by this factor.

The prevalence of pPOLOS and the strength of its association with complications vary widely depending on the pPOLOS definition [33]. There is no standard definition of pPOLOS: > 50^th^ percentile [13], > 75^th^ percentile [11], and > 90^th^ percentile [14]. In our cohort, we chose quartiles of surgery duration in each surgical type that provided larger comparable groups. The comparison groups vary as well: entire population [34], by type of surgery [13], by surgical procedures [35].

### Strengths and limitations

This study has several notable strengths. First, the large patient cohort permitted inclusion of various surgical types, enhancing analytical robustness. The inclusion and non-inclusion criteria used define a homogeneous patient population. Another strength is that the outcome, pPOLOS, is easy to find and has no missing data. The proposed method offers a dual perspective: it not only identifies key variables but also uncovers families of variables, potentially bridging diverse research efforts focusing on similar clinical outcomes but different biomarkers. Focusing exclusively on blood pressure variables, this approach reveals clusters linked with pPOLOS. However, further studies should address the impact of confounding factors on pPOLOS. Interestingly, it is plausible that important features such as age or comorbidities, like hypertension, could be encompassed within certain clusters identified by this method, a work beyond the scope of this study.

Our study focused on three IOH features selected by an unsupervised clustering method. This selection method for features tends to be more objective and represents the strength of our work. The high number of patients included, and the completeness of the pPOLOS collection are also main strengths.

Our study has several limits. First, important methodological limitations mandate cautious interpretation of pPOLOS. Analyses should adjust for clinical and social determinants [10], incorporate relevant contextual factors (including institutional practices and discharge pathways), and explicitly account for readmissions related to the index surgery rather than omitting them. However, higher-threshold definitions such as pPOLOS^75^ [11] and pPOLOS^90^ [36] are probably less sensitive to these biases than pPOLOS^50^. Finally, replacement of pPOLOS by the number of days spent at home during the first 30 days after surgery has been proposed as a more patient-centered outcome measure [37]. Second, major confounders (including ASA status, comorbidities, and baseline blood pressure) were not adjusted for in this analysis. Consequently, these associative findings may reflect underlying patient frailty and specific baseline characteristics rather than a direct causal effect of intraoperative blood pressure variation. Third, POLOS was not adjusted for age, sex, or hypertension status in the primary analysis. However, we addressed these factors through stratified subgroup analyses and threshold assessments, which revealed important differences by age and sex. Fourth, we examined only absolute IOH thresholds rather than relative changes from baseline blood pressure. Selection of an appropriate baseline BP remains challenging. First operating room BP was the only available reading in our cohort, and it is recognized as a poor surrogate for true baseline BP [38]. In our opinion, this also represents a strength because of its ease of application. Finally, data on specific postoperative organ dysfunction and complications were not available in our cohort.

### Generalizability

Our findings may be difficult to generalize as this was a single-center study with a predominance of neurosurgical and digestive surgery patients (no cardiac surgery included), and a large proportion of patients received total intravenous anesthesia. We employed simple, clinically available variables (MinMAP, DropPP) and an objective outcome (pPOLOS), which should facilitate reproducibility and clinical implementation. The Perioperative Quality Initiative guidelines recommend an IOH MAP threshold between 60–70 mmHg [7]. This recommendation applies to a general population without any consideration of age, sex, or medical history. However, neurosurgical patients, particularly those who have undergone surgery for an intracranial condition, may have impaired cerebral autoregulation, requiring blood pressure targets to be adjusted accordingly. Therefore, our findings cannot be generalized to this patient population. We believe that future hemodynamic management strategies should incorporate patient-specific characteristics including age, sex, and comorbidity status. PP is an underappreciated hemodynamic variable that warrants integration into IOH diagnostic and therapeutic algorithms. Few studies have focused on IOH with an interventional design. Futier et al. showed that strict systolic blood pressure management can improve postoperative organ dysfunction [39]. This work sets the foundation for causality between IOH and organ injury.

## Conclusion

This study demonstrates that DropPP is a robust pPOLOS risk predictor that remains consistent across different pPOLOS definitions, while MinMAP provides complementary prognostic information with age- and sex-specific thresholds. Future interventional studies should investigate IOH management strategies using multiple thresholds based on multiple hemodynamic variables, particularly DropPP and MinMAP, tailored to patient characteristics. Integration of these hemodynamic variables with patient-specific characteristics may help refine the definition of clinically significant intraoperative hypotension and establish personalized blood pressure targets [40].

## Supporting information

S1 Appendix Illustration of spline model selection and trend characterization. (DOCX)
