# Supplementary material for: Association of intraoperative pulse pressure drop and minimum mean arterial pressure with postoperative length of stay: A stratified reanalysis of published data by age and sex
Source: PLoS One. 2026 May 28;21(5):e0350048. doi: 10.1371/journal.pone.0350048 (PMC13218502; doi:10.1371/journal.pone.0350048)
Supplement: S1 Appendix — (DOCX) [file pone.0350048.s001.docx]

**S1 Appendix**

**Illustration of spline model selection and trend characterization**.


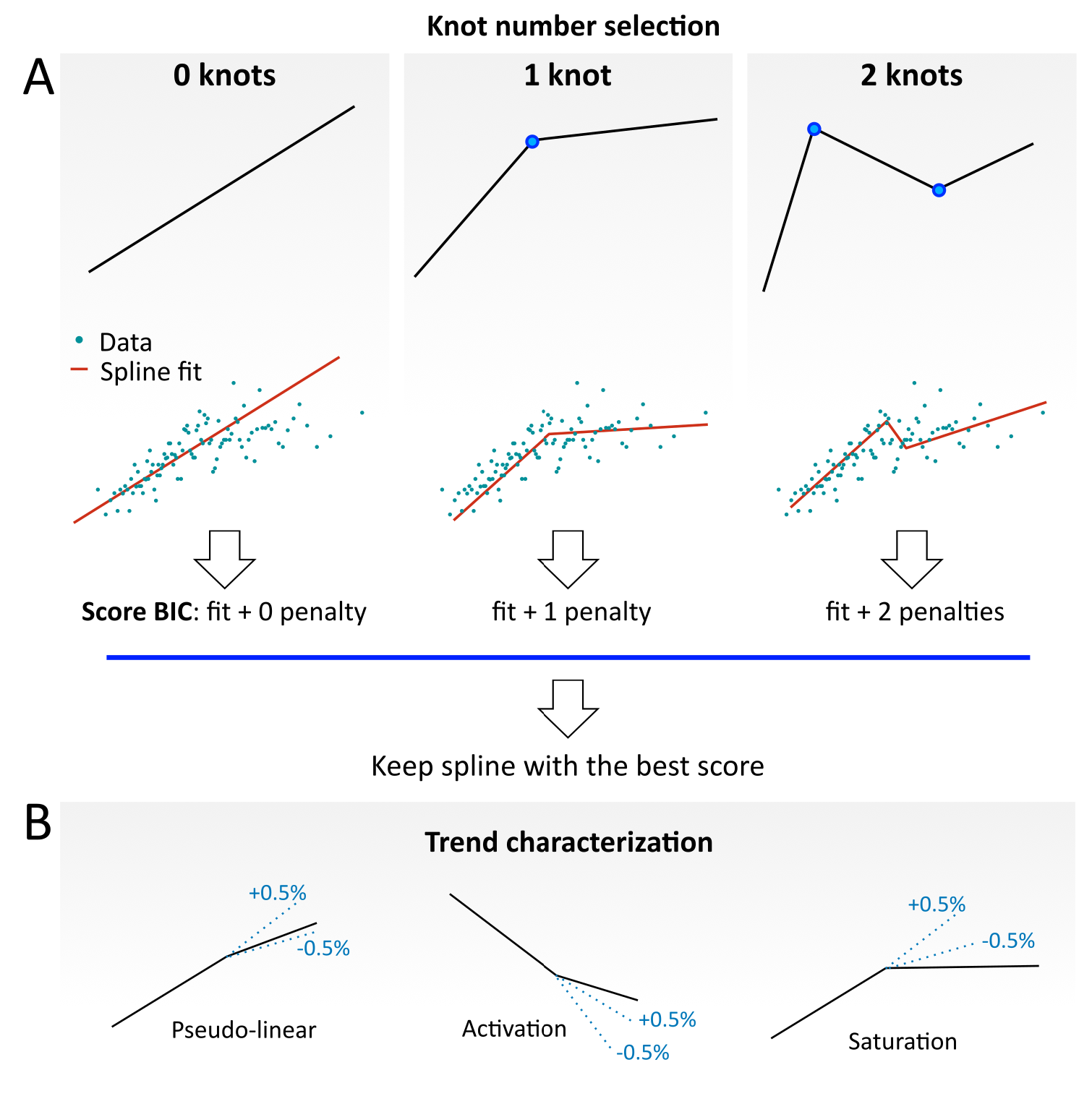


**A.** Knot number selection for the piecewise linear, continuous spline used to model the relationship between a BP-derived predictor and PLOS risk. Candidate models with 0 knots (simple linear fit), one knot, or two knots were fitted (blue dots indicate knot locations). Observations are shown as blue points and the fitted spline as a red line. Model comparison was performed using the BIC, which combines goodness of fit (mentioned as fit) with a complexity penalty that increases with the number of estimated parameters (Penalty). The spline model retained for each predictor was the one with the lowest BIC.

**B.** Trend characterization based on the change in slope between adjacent spline segments at the knot. When the slope change remains within ±0.5 (% risk per unit of predictor), the relationship is classified as pseudo-linear. A slope increase greater than +0.5 indicates an activation pattern, whereas a slope decrease less than −0.5 indicates a saturation pattern.
