# Supplementary material for: Association of intraoperative pulse pressure drop and minimum mean arterial pressure with postoperative length of stay: A stratified reanalysis of published data by age and sex
Source: PLoS One. 2026 May 28;21(5):e0350048. doi: 10.1371/journal.pone.0350048 (PMC13218502; doi:10.1371/journal.pone.0350048)
Supplement: Attachment — Submitted filename: renamed_dd04d.docx [file pone.0350048.s002.docx]

We would like to thank the reviewer for their insightful comments, which have led us to clarify certain points. We have also improved clarity and flow as suggested by the reviewer. Changes are shown in red and crossed out (suppression) and in blue (addition) in the marked file.

**Major comment 1: Outcome definition and clinical interpretability**
The definition of pPOLOS as length of stay above the median (and higher percentiles) is methodologically reasonable but remains difficult to interpret clinically. The authors should more clearly justify this choice and explicitly discuss how this surrogate relates to concrete postoperative morbidity. A short paragraph clarifying the strengths and limitations of pPOLOS as a proxy outcome, particularly in the absence of direct complication data, would substantially improve interpretability.

**Response to major comment 1**

Indeed, although we discussed the limitations of pPOLOS in the initial version, it is fair to be more specific about its limitations.

• We modified lines 51 to 56 of the initial version (Introduction section) by adding in particular a reference to the work of Newgard et al. who showed that pPOLOS is a surrogate for organ injury in a cohort of severely injured patients (lines 50 to 56 of the revised version): “Postoperative length of stay (POLOS) represents an objective, readily ascertainable outcome available for all surgical patients. While POLOS is strongly influenced by severe postoperative complications, it is also substantially affected by non-clinical factors, particularly social determinants [4], making it a potential surrogate endpoint for cumulative organ injury as demonstrated in a cohort of severely injured patients [5]. Currently, the relationship between IOH and POLOS remains understudied and has primarily been examined as a secondary outcome [6]. Further investigation is required to elucidate the magnitude and clinical relevance of this association.”

• We added a paragraph in the Discussion section (lines 297 to 304): “POLOS functions as a pragmatic surrogate for complications in large-scale analyses, capturing objective data that circumvent underreporting biases [10]. Nevertheless, its validity is compromised by multiple confounding factors: institutional discharge practices, social determinants, logistical constraints, and financial pressures [11, 12]. Additionally, enhanced recovery after surgery protocols reduce length of stay while obscuring post-discharge morbidity, further dissociating PLOS from true surgical morbidity. Finally, this metric lacks clinical specificity regarding complication type and severity, prevents causal inference, and suffers from definitional heterogeneity across studies (> 50th percentile [13], > 75th percentile [11] and > 90th percentile [14]), thereby limiting comparability.”

• We added a paragraph in the Strengths and limitations section (lines 402 to 409): “First, important methodological limitations mandate cautious interpretation of pPOLOS. Analyses should adjust for clinical and social determinants [10], incorporate relevant contextual factors (including institutional practices and discharge pathways), and explicitly account for readmissions related to the index surgery rather than omitting them. However, higher-threshold definitions such as pPOLOS^75^ [11] and pPOLOS^90^ [36] are probably less sensitive to these biases than pPOLOS^50^. Finally, replacement of pPOLOS by the number of days spent at home during the first 30 days after surgery has been proposed as a more patient-centered outcome measure [37].”

**Major comment 2: Confounding and adjustment strategy**
While subgroup analyses by age and sex are performed, the absence of multivariable adjustment for major confounders (e.g., ASA status, comorbidities, hypertension, type of surgery, anesthesia technique) is a significant limitation. Even if additional adjustment is not feasible, this limitation should be more clearly acknowledged and discussed. The authors should explicitly state that observed associations may partly reflect patient frailty rather than causal effects of intraoperative blood pressure variables.

**Response to major comment 2**

We agree that the absence of multivariable adjustment for key confounders such as ASA or hypertension status, is an significant limitation. Our spline procedure was designed to characterize the functional form of the univariate association between each intraoperative blood pressure variable and PLOS risk. We therefore treated the age and sex subgroup analyses as descriptive heterogeneity checks, not as confounding control. An adjusted model combining spline terms with multiple covariates could be considered, but data-driven knot selection may become unstable and the added flexibility increases the risk of overfitting. Propensity-based approaches are another option, but they would likely face limited overlap (positivity) across covariate profiles and instability in the tails of the exposure distribution. We have revised the manuscript to state explicitly your remark that the reported associations are non-causal and may partly reflect underlying patient frailty and procedural complexity rather than a direct effect of intraoperative blood pressure.

We have suppressed the sentence (“First, the retrospective design did not allow us to establish any causality between IOH features and pPOLOS risk. It certainly exposed frailty.“; lines 413 to 415) from our initial manuscript and replace it by the following sentence (lines 409 to 412): “Second, major confounders (including ASA status, comorbidities, and baseline blood pressure) were not adjusted for in this analysis. Consequently, these associative findings may reflect underlying patient frailty and specific baseline characteristics rather than a direct causal effect of intraoperative blood pressure variation.“

**Major comment 3:** **Pulse pressure–based variables**
DropPP and CumTimePP>61 mmHg are interesting and relatively novel metrics. However, their physiological and clinical interpretation requires further clarification. In particular, the rationale for the 61 mmHg threshold should be more explicitly justified, and the potential overlap with arterial stiffness or chronic hypertension should be discussed more cautiously to avoid overinterpretation.

**Response to** **major comment 3:**

As we wrote in the Introduction section, our team showed in a precedent study that three intraoperative IOH variables are related to prolonged pPOLOS when pPOLOS is defined as > 50^th^ percentile: drop in pulse pressure (DropPP, difference between maximal value and minimal value of intraoperative PP), minimal value of mean arterial pressure (MinMAP), and CumTimePP>61mmHg (cumulative time of PP spent over 61 mmHg per hour of surgery).

The methodological approach was distinctive in use of an unsupervised clustering method. Note that the limit of 61 mmHg was found by statistical analysis and was not chosen by the authors. These novel hemodynamic metrics are particularly noteworthy, particularly DropPP which is the sole variable associated with pPOLOS under its most stringent definition (pPOLOS^90^).

We wrote in the initial manuscript: “Pulse pressure is an important blood pressure variable in hemodynamic evaluation which is related to both stroke volume [9] and arterial stiffness [10]. A low PP may reflect hypovolemia or impaired left ventricular systolic function and conversely a high PP suggests a reduced arterial compliance.” This was too simplistic, and in the revised version we offer a more comprehensive description of the pathophysiology of PP and its clinical implications.

To respond to the Reviewer’s comment,

• We have added a brief summary of PP physiopathology in the Discussion section (lines 306 to 314): “Pulse pressure represents the pulsatile component of the blood pressure curve. It arises from the interaction between cardiac ejection and the elastic properties of the arterial circulation [15] and its change is proportional to volume change but inversely proportional to arterial compliance. Consequently, several pathological conditions result in widened PP particularly: aortic arteriosclerosis, since stiffening of the aorta and large arteries reduced arterial compliance and alter wave reflection [15]. From this perspective, it should be noted that the average age of our patients is 55.0 ± 17.5 years and that 13.9% are classified as having high blood pressure [8]. On the other hand, narrow PP occurs in conditions characterized by reduced stroke volume or cardiac output [16]. PP should not be confused with arterial pulse pressure variation with mechanical ventilation [17].”

• The 61 mmHg limit is explained in the manuscript by referencing our previous publication.

The features derived from the cumulative time with PP >61 mmHg were obtained using a data-driven approach [reference n°8: Cartailler J, Beaucote V, Trillat B, Gayat E, Le Guen M, Vallee A, et al. Deciphering the explanatory potential of blood pressure variables on post-operative length of stay through hierarchical clustering: A retrospective monocentric study. PLoS One. 2024;19(9):e0308910. doi: 10.1371/journal.pone.0308910]. In this prior work, we developed a method that identified this metric as a strong representative of clusters that were complementary to those captured by DropPP and MinMAP.

**Major comment 4: Spline methodology and thresholds**
The spline-based approach is a strength, but the description of knot selection and threshold definition is dense and may be difficult for non-statistical readers. A simplified explanation in the Methods or a short schematic explanation in the Supplementary Material would improve clarity. Additionally, the clinical meaning of “activation” versus “pseudo-linear” relationships should be more clearly articulated.

**Response to** **major comment 4:** Thank you for this helpful comment regarding the clarity of the presented methodology. We agree that this section could be clearer, and we have revised the paragraph accordingly to improve readability and precision.
The previous section Methods (lines 134 to 150) is replaced with: “To model the association between PLOS risk and each BP variable, we fitted piecewise linear continuous splines with 0 (purely linear), 1, or 2 knots (S1 Appendix, panel A). Model selection relied on the Bayesian Information Criterion (BIC), defined as BIC = −2 log L + p ln(n), where L is the likelihood, p is the number of estimated parameters, and n is the sample size, thereby balancing goodness of fit and model complexity. For the 1-knot and 2-knot candidates, knot locations were chosen by an exhaustive grid search over the predictor scale: the search domain was centered on the population median and bounded by ±2 standard deviations, with a resolution of 1 unit (e.g., 1 mmHg for MinMAP and DropPP). For 2-knot models, all ordered pairs in this grid were evaluated. For each variable (DropPP, MinMAP, and CumTimePP>61 mmHg), the final model (0, 1, or 2 knots) was the candidate with the lowest BIC. After selecting the model, we characterized the fitted pattern as pseudo-linear, activation, or saturation based on the change in slope between adjacent segments at the knot. Specifically, pseudo-linearity was defined when the absolute slope change lay between −0.5 and +0.5 % per mmHg (or % per min); slope increases greater than +0.5 were classified as activation, whereas decreases less than −0.5 were classified as saturation (S1 Appendix, panel B). This procedure was applied to the full cohort and to subgroups (women, men, age <65 years, and age ≥65 years).”

S1 Appendix was upload with revised version.

**Major comment 5: Generalizability**
Given the single-center design, surgical case mix, and high proportion of specific anesthesia techniques, the external validity of the findings is uncertain. This should be more clearly emphasized in the Discussion.

**Response to** **major comment 5**

We have created a section entitled "Generalizability" to give greater visibility to this issue. Our text was modified to include the reviewer's comment (lines 422 to 424): "Our findings may be difficult to generalize as this was a single-center study with a predominance of neurosurgical and digestive surgery patients (no cardiac surgery included), and a large proportion of patients received total intravenous anesthesia."

**Minor comment 1:**

Some figures (especially multi-panel spline figures) are visually dense; clearer labeling or simplified legends would help readability.

**Response to minor comment 1:**

We believe that the figures should be kept as they are, but we have simplified their captions. Modifying the figures would result in a significant increase in their number.

**Minor comment 2:**

Minor language polishing is recommended to improve clarity and flow.

**Response to minor comment 2:**

We have reviewed the entire manuscript and made numerous changes that should address this recommendation.
