# Supplementary material for: Association of intraoperative pulse pressure drop and minimum mean arterial pressure with postoperative length of stay: A stratified reanalysis of published data by age and sex
Source: PLoS One. 2026 May 28;21(5):e0350048. doi: 10.1371/journal.pone.0350048 (PMC13218502; doi:10.1371/journal.pone.0350048)
Supplement: Attachment — Submitted filename: renamed_e838f.docx [file pone.0350048.s003.docx]

PONE-D-25-54481R1

Association of intraoperative pulse pressure drop and minimum mean arterial pressure with postoperative length of stay: A stratified reanalysis of published data by age and sex

PLOS One

We would like to thank the reviewer for its comments, which have led us to clarify or modify certain points. are shown in red and crossed out (suppression) and in blue (addition) in the marked file.

**Minor comment 1:** Speculative interpretation of CumTimePP >61 mmHg

In the Discussion (lines 334–345), the authors hypothesize that CumTimePP >61 mmHg reflects hypertensive status based on indirect reasoning. While this is an interesting hypothesis, the language should be made more explicitly conditional throughout this paragraph. Phrases such as "we can assume" and "could represent the hypertensive population" would benefit from further hedging, and the authors should acknowledge that this interpretation remains unverified in the absence of direct adjustment for known hypertensive patients in the analysis.

**Response to minor comment 1:** The reviewer is right to suggest that the text be written more cautiously. This has led to a revision of this paragraph: “In our study, a longer time spent above 61 mmHg of PP was associated with increased pPOLOS. The maximum risk of pPOLOS was reached quickly, mainly after the first two minute per hour of surgery. This pattern suggests a separation between patients with short versus prolonged exposure to elevated PP, which might correspond to non-hypertensive and hypertensive profiles, respectively. However, the hypothesis that CumTimePP > 61 mmHg reflects underlying hypertensive status remains speculative, as we did not directly adjust for or stratify by documented hypertension in this analysis, and should therefore be interpreted with caution and confirmed in future studies.”

**Minor comment 2:** Neurosurgical patient specificity in the Generalizability section

The Generalizability section notes the predominance of neurosurgical patients but does not discuss the implications. Neurosurgical patients represent the single largest subgroup (n=2,062) and are physiologically distinct with respect to cerebrovascular autoregulation and blood pressure targets. The authors should briefly acknowledge that intraoperative blood pressure thresholds in neurosurgical patients may differ from the general surgical population, which further limits generalizability beyond what is currently stated.

**Response to minor comment 2:** We would like to thank the reviewer for this very pertinent comment. It prompted us to add a sentence to this chapter that addresses it: “… However, neurosurgical patients, particularly those who have undergone surgery for an intracranial condition, may have impaired cerebral autoregulation, requiring blood pressure targets to be adjusted accordingly. Therefore, our findings cannot be generalized to this patient population….”

**Minor comment 3:** Typographical error in the text

In the Generalizability section, reference 39 is cited as "Flutier et al." whereas the reference list correctly identifies the first author as "Futier." This should be corrected for consistency.

**Response to minor comment 3:** We apologize for this typographical error, which has been corrected.
