# Supplementary material for: Association of intraoperative pulse pressure drop and minimum mean arterial pressure with postoperative length of stay: A stratified reanalysis of published data by age and sex
Source: PLoS One. 2026 May 28;21(5):e0350048. doi: 10.1371/journal.pone.0350048 (PMC13218502; doi:10.1371/journal.pone.0350048)
Supplement: Peer review history

# Peer review: Decision Letter 0

**DOI:** 10.1371/journal.pone.0350048.r001

**Authors:** Benjamin Benzon

8 Jan 2026

PONE-D-25-54481

Association of intraoperative pulse pressure drop and minimum mean arterial pressure with postoperative length of stay: A stratified reanalysis of published data by age and sex

PLOS One

Dear Dr. Fischler,

Thank you for submitting your manuscript to PLOS ONE. After careful consideration, we feel that it has merit but does not fully meet PLOS ONE’s publication criteria as it currently stands. Therefore, we invite you to submit a revised version of the manuscript that addresses the points raised during the review process.

==============================

Dear authors reviewers made some comments that make sense, please address them.

==============================

Please submit your revised manuscript by Feb 22 2026 11:59PM. If you will need more time than this to complete your revisions, please reply to this message or contact the journal office at plosone@plos.org. When you're ready to submit your revision, log on to https://www.editorialmanager.com/pone/ and select the 'Submissions Needing Revision' folder to locate your manuscript file.

Please include the following items when submitting your revised manuscript:

- A letter that responds to each point raised by the academic editor and reviewer(s). You should upload this letter as a separate file labeled 'Response to Reviewers'.
- A marked-up copy of your manuscript that highlights changes made to the original version. You should upload this as a separate file labeled 'Revised Manuscript with Track Changes'.
- An unmarked version of your revised paper without tracked changes. You should upload this as a separate file labeled 'Manuscript'.

If you would like to make changes to your financial disclosure, please include your updated statement in your cover letter. Guidelines for resubmitting your figure files are available below the reviewer comments at the end of this letter.

If applicable, we recommend that you deposit your laboratory protocols in protocols.io to enhance the reproducibility of your results. Protocols.io assigns your protocol its own identifier (DOI) so that it can be cited independently in the future. For instructions see: https://journals.plos.org/plosone/s/submission-guidelines#loc-laboratory-protocols. Additionally, PLOS ONE offers an option for publishing peer-reviewed Lab Protocol articles, which describe protocols hosted on protocols.io. Read more information on sharing protocols at https://plos.org/protocols?utm_medium=editorial-email&utm_source=authorletters&utm_campaign=protocols.

We look forward to receiving your revised manuscript.

Kind regards,

Benjamin Benzon, Ph.D., M.D.

Academic Editor

PLOS One

Journal Requirements:

When submitting your revision, we need you to address these additional requirements.

1. Please ensure that your manuscript meets PLOS ONE's style requirements, including those for file naming. The PLOS ONE style templates can be found at

https://journals.plos.org/plosone/s/file?id=wjVg/PLOSOne_formatting_sample_main_body.pdf and

https://journals.plos.org/plosone/s/file?id=ba62/PLOSOne_formatting_sample_title_authors_affiliations.pdf

2. If the reviewer comments include a recommendation to cite specific previously published works, please review and evaluate these publications to determine whether they are relevant and should be cited. There is no requirement to cite these works unless the editor has indicated otherwise.

[Note: HTML markup is below. Please do not edit.]

Reviewers' comments:

Reviewer's Responses to Questions

**Comments to the Author**

1. Is the manuscript technically sound, and do the data support the conclusions?

The manuscript must describe a technically sound piece of scientific research with data that supports the conclusions. Experiments must have been conducted rigorously, with appropriate controls, replication, and sample sizes. The conclusions must be drawn appropriately based on the data presented.

Reviewer #1: Partly

Reviewer #2: Partly

**********

2. Has the statistical analysis been performed appropriately and rigorously?

Reviewer #1: Yes

Reviewer #2: I Don't Know

**********

3. Have the authors made all data underlying the findings in their manuscript fully available?

The PLOS Data policy requires authors to make all data underlying the findings described in their manuscript fully available without restriction, with rare exception (please refer to the Data Availability Statement in the manuscript PDF file). The data should be provided as part of the manuscript or its supporting information, or deposited to a public repository. For example, in addition to summary statistics, the data points behind means, medians and variance measures should be available. If there are restrictions on publicly sharing data—e.g. participant privacy or use of data from a third party—those must be specified.

Reviewer #1: Yes

Reviewer #2: No

**********

4. Is the manuscript presented in an intelligible fashion and written in standard English?

PLOS ONE does not copyedit accepted manuscripts, so the language in submitted articles must be clear, correct, and unambiguous. Any typographical or grammatical errors should be corrected at revision, so please note any specific errors here.

Reviewer #1: Yes

Reviewer #2: Yes

**********

5. Review Comments to the Author

Please use the space provided to explain your answers to the questions above. You may also include additional comments for the author, including concerns about dual publication, research ethics, or publication ethics. (Please upload your review as an attachment if it exceeds 20,000 characters)

Reviewer #1: Dear Authors,

Thank you for the opportunity to review your manuscript entitled “Public Interest and Surgical Behavior in Breast Implant Illness: Correlation Between Google Trends and Breast Implant Removal Rates in Colombia.” Overall, the study is well conceived and clearly presented. The idea of correlating public search interest with surgical statistics is innovative and has potential implications for understanding patient awareness and healthcare demand. The introduction provides good context, and the discussion links findings to prior literature. The major improvements needed relate to methodological clarity, statistical transparency, and tightening of the discussion to avoid causal interpretations.

Major Comments

1. Definition and Selection of Search Terms

Please expand on the criteria used to define and group the Spanish search terms. Because Spanish has many colloquial variants, a short explanation of how synonyms, plural forms, or related expressions were handled would improve reproducibility. Clarify whether misspelled terms or slang were considered and why they were excluded.

2. Temporal Alignment of Data Sources

The Google Trends dataset covers 2011–2025, while ISAPS data span 2016–2023. Explicitly state that correlations were computed only for the overlapping years and clarify how partial data from 2025 were handled. The captions of Figures 3–4 should also specify the analyzed period.

3. Statistical Transparency

Pearson and Spearman correlations are appropriate, but please report the number of data points (years = 8) and degrees of freedom. Confidence intervals or time-lag analyses would strengthen the argument that public interest precedes procedural changes rather than merely co-occurring with them.

4. Interpretation and Causality

Some sentences imply that increased online interest influenced the rise in explantations. Correlation does not establish causation; therefore, rephrase these statements more cautiously (e.g., “is associated with” or “coincided with”). If feasible, consider including a brief lagged-correlation analysis to test whether search trends anticipate surgical rates.

5. Figures and Tables

Figures are informative but could benefit from improved readability: enlarge axis labels, unify font size, and ensure consistent color schemes. In Table 1, correct the typo “Breas implant removal.” Adding a brief description of the number of years analyzed would aid interpretation.

6. Limitations Section

Expand the limitations to emphasize that Google Trends provides relative, not absolute, search volumes; Spanish-language diversity may bias representation; ISAPS data include only participating surgeons; and potential confounders (media coverage, socioeconomic shifts, regulatory changes, or pandemic effects) may affect both online behavior and surgical demand.

7. Discussion and References

Strengthen the discussion by comparing your findings with other regional or Latin-American analyses of aesthetic-surgery trends, if available. Avoid repetition between the first and third discussion paragraphs, both describing the impact of media coverage.

Reviewer #2: This article is too complicated for simple correlation between blood pressure and lenght of hospital stay.

In first place it is difficult to establish direct correlation as lenght of stay is multifacotirial. For me this is statistical cherry picking.

**********

6. PLOS authors have the option to publish the peer review history of their article (what does this mean?). If published, this will include your full peer review and any attached files.

If you choose “no”, your identity will remain anonymous but your review may still be made public.

**Do you want your identity to be public for this peer review?** For information about this choice, including consent withdrawal, please see our Privacy Policy.

Reviewer #1: No

Reviewer #2: No

**********

[NOTE: If reviewer comments were submitted as an attachment file, they will be attached to this email and accessible via the submission site. Please log into your account, locate the manuscript record, and check for the action link "View Attachments". If this link does not appear, there are no attachment files.]

To ensure your figures meet our technical requirements, please review our figure guidelines: https://journals.plos.org/plosone/s/figures

You may also use PLOS’s free figure tool, NAAS, to help you prepare publication quality figures: https://journals.plos.org/plosone/s/figures#loc-tools-for-figure-preparation.

NAAS will assess whether your figures meet our technical requirements by comparing each figure against our figure specifications.

# Author comment: Author response to Decision Letter 1

**DOI:** 10.1371/journal.pone.0350048.r002

1 Mar 2026

We would like to thank the reviewer for their insightful comments, which have led us to clarify certain points. We have also improved clarity and flow as suggested by the reviewer. Changes are shown in red and crossed out (suppression) and in blue (addition) in the marked file.

Major comment 1: Outcome definition and clinical interpretability

The definition of pPOLOS as length of stay above the median (and higher percentiles) is methodologically reasonable but remains difficult to interpret clinically. The authors should more clearly justify this choice and explicitly discuss how this surrogate relates to concrete postoperative morbidity. A short paragraph clarifying the strengths and limitations of pPOLOS as a proxy outcome, particularly in the absence of direct complication data, would substantially improve interpretability.

Response to major comment 1

Indeed, although we discussed the limitations of pPOLOS in the initial version, it is fair to be more specific about its limitations.

• We modified lines 51 to 56 of the initial version (Introduction section) by adding in particular a reference to the work of Newgard et al. who showed that pPOLOS is a surrogate for organ injury in a cohort of severely injured patients (lines 50 to 56 of the revised version): “Postoperative length of stay (POLOS) represents an objective, readily ascertainable outcome available for all surgical patients. While POLOS is strongly influenced by severe postoperative complications, it is also substantially affected by non-clinical factors, particularly social determinants [4], making it a potential surrogate endpoint for cumulative organ injury as demonstrated in a cohort of severely injured patients [5]. Currently, the relationship between IOH and POLOS remains understudied and has primarily been examined as a secondary outcome [6]. Further investigation is required to elucidate the magnitude and clinical relevance of this association.”

• We added a paragraph in the Discussion section (lines 297 to 304): “POLOS functions as a pragmatic surrogate for complications in large-scale analyses, capturing objective data that circumvent underreporting biases [10]. Nevertheless, its validity is compromised by multiple confounding factors: institutional discharge practices, social determinants, logistical constraints, and financial pressures [11, 12]. Additionally, enhanced recovery after surgery protocols reduce length of stay while obscuring post-discharge morbidity, further dissociating PLOS from true surgical morbidity. Finally, this metric lacks clinical specificity regarding complication type and severity, prevents causal inference, and suffers from definitional heterogeneity across studies (> 50th percentile [13], > 75th percentile [11] and > 90th percentile [14]), thereby limiting comparability.”

• We added a paragraph in the Strengths and limitations section (lines 402 to 409): “First, important methodological limitations mandate cautious interpretation of pPOLOS. Analyses should adjust for clinical and social determinants [10], incorporate relevant contextual factors (including institutional practices and discharge pathways), and explicitly account for readmissions related to the index surgery rather than omitting them. However, higher-threshold definitions such as pPOLOS75 [11] and pPOLOS90 [36] are probably less sensitive to these biases than pPOLOS50. Finally, replacement of pPOLOS by the number of days spent at home during the first 30 days after surgery has been proposed as a more patient-centered outcome measure [37].”

Major comment 2: Confounding and adjustment strategy

While subgroup analyses by age and sex are performed, the absence of multivariable adjustment for major confounders (e.g., ASA status, comorbidities, hypertension, type of surgery, anesthesia technique) is a significant limitation. Even if additional adjustment is not feasible, this limitation should be more clearly acknowledged and discussed. The authors should explicitly state that observed associations may partly reflect patient frailty rather than causal effects of intraoperative blood pressure variables.

Response to major comment 2

We agree that the absence of multivariable adjustment for key confounders such as ASA or hypertension status, is an significant limitation. Our spline procedure was designed to characterize the functional form of the univariate association between each intraoperative blood pressure variable and PLOS risk. We therefore treated the age and sex subgroup analyses as descriptive heterogeneity checks, not as confounding control. An adjusted model combining spline terms with multiple covariates could be considered, but data-driven knot selection may become unstable and the added flexibility increases the risk of overfitting. Propensity-based approaches are another option, but they would likely face limited overlap (positivity) across covariate profiles and instability in the tails of the exposure distribution. We have revised the manuscript to state explicitly your remark that the reported associations are non-causal and may partly reflect underlying patient frailty and procedural complexity rather than a direct effect of intraoperative blood pressure.

We have suppressed the sentence (“First, the retrospective design did not allow us to establish any causality between IOH features and pPOLOS risk. It certainly exposed frailty.“; lines 413 to 415) from our initial manuscript and replace it by the following sentence (lines 409 to 412): “Second, major confounders (including ASA status, comorbidities, and baseline blood pressure) were not adjusted for in this analysis. Consequently, these associative findings may reflect underlying patient frailty and specific baseline characteristics rather than a direct causal effect of intraoperative blood pressure variation.“

Major comment 3: Pulse pressure–based variables

DropPP and CumTimePP>61 mmHg are interesting and relatively novel metrics. However, their physiological and clinical interpretation requires further clarification. In particular, the rationale for the 61 mmHg threshold should be more explicitly justified, and the potential overlap with arterial stiffness or chronic hypertension should be discussed more cautiously to avoid overinterpretation.

Response to major comment 3:

As we wrote in the Introduction section, our team showed in a precedent study that three intraoperative IOH variables are related to prolonged pPOLOS when pPOLOS is defined as > 50th percentile: drop in pulse pressure (DropPP, difference between maximal value and minimal value of intraoperative PP), minimal value of mean arterial pressure (MinMAP), and CumTimePP>61mmHg (cumulative time of PP spent over 61 mmHg per hour of surgery).

The methodological approach was distinctive in use of an unsupervised clustering method. Note that the limit of 61 mmHg was found by statistical analysis and was not chosen by the authors. These novel hemodynamic metrics are particularly noteworthy, particularly DropPP which is the sole variable associated with pPOLOS under its most stringent definition (pPOLOS90).

We wrote in the initial manuscript: “Pulse pressure is an important blood pressure variable in hemodynamic evaluation which is related to both stroke volume [9] and arterial stiffness [10]. A low PP may reflect hypovolemia or impaired left ventricular systolic function and conversely a high PP suggests a reduced arterial compliance.” This was too simplistic, and in the revised version we offer a more comprehensive description of the pathophysiology of PP and its clinical implications.

To respond to the Reviewer’s comment,

• We have added a brief summary of PP physiopathology in the Discussion section (lines 306 to 314): “Pulse pressure represents the pulsatile component of the blood pressure curve. It arises from the interaction between cardiac ejection and the elastic properties of the arterial circulation [15] and its change is proportional to volume change but inversely proportional to arterial compliance. Consequently, several pathological conditions result in widened PP particularly: aortic arteriosclerosis, since stiffening of the aorta and large arteries reduced arterial compliance and alter wave reflection [15]. From this perspective, it should be noted that the average age of our patients is 55.0 ± 17.5 years and that 13.9% are classified as having high blood pressure [8]. On the other hand, narrow PP occurs in conditions characterized by reduced stroke volume or cardiac output [16]. PP should not be confused with arterial pulse pressure variation with mechanical ventilation [17].”

• The 61 mmHg limit is explained in the manuscript by referencing our previous publication.

The features derived from the cumulative time with PP >61 mmHg were obtained using a data-driven approach [reference n°8: Cartailler J, Beaucote V, Trillat B, Gayat E, Le Guen M, Vallee A, et al. Deciphering the explanatory potential of blood pressure variables on post-operative length of stay through hierarchical clustering: A retrospective monocentric study. PLoS One. 2024;19(9):e0308910. doi: 10.1371/journal.pone.0308910]. In this prior work, we developed a method that identified this metric as a strong representative of clusters that were complementary to those captured by DropPP and MinMAP.

Major comment 4: Spline methodology and thresholds

The spline-based approach is a strength, but the description of knot selection and threshold definition is dense and may be difficult for non-statistical readers. A simplified explanation in the Methods or a short schematic explanation in the Supplementary Material would improve clarity. Additionally, the clinical meaning of “activation” versus “pseudo-linear” relationships should be more clearly articulated.

Response to major comment 4: Thank you for this helpful comment regarding the clarity of the presented methodology. We agree that this section could be clearer, and we have revised the paragraph accordingly to improve readability and precision.

The previous section Methods (lines 134 to 150) is replaced with: “To model the association between PLOS risk and each BP variable, we fitted piecewise linear continuous splines with 0 (purely linear), 1, or 2 knots (S1 Appendix, panel A). Model selection relied on the Bayesian Information Criterion (BIC), defined as BIC = −2 log L + p ln(n), where L is the likelihood, p is the number of estimated parameters, and n is the sample size, thereby balancing goodness of fit and model complexity. For the 1-knot and 2-knot candidates, knot locations were chosen by an exhaustive grid search over the predictor scale: the search domain was centered on the population median and bounded by ±2 standard deviations, with a resolution of 1 unit (e.g., 1 mmHg for MinMAP and DropPP). For 2-knot models, all ordered pairs in this grid were evaluated. For each variable (DropPP, MinMAP, and CumTimePP>61 mmHg), the final model (0, 1, or 2 knots) was the candidate with the lowest BIC. After selecting the model, we characterized the fitted pattern as pseudo-linear, activation, or saturation based on the change in slope between adjacent segments at the knot. Specifically, pseudo-linearity was defined when the absolute slope change lay between −0.5 and +0.5 % per mmHg (or % per min); slope increases greater than +0.5 were classified as activation, whereas decreases less than −0.5 were classified as saturation (S1 Appendix, panel B). This procedure was applied to the full cohort and to subgroups (women, men, age <65 years, and age ≥65 years).”

S1 Appendix was upload with revised version.

Major comment 5: Generalizability

Given the single-center design, surgical case mix, and high proportion of specific anesthesia techniques, the external validity of the findings is uncertain. This should be more clearly emphasized in the Discussion.

Response to major comment 5

We have created a section entitled "Generalizability" to give greater visibility to this issue. Our text was modified to include the reviewer's comment (lines 422 to 424): "Our findings may be difficult to generalize as this was a single-center study with a predominance of neurosurgical and digestive surgery patients (no cardiac surgery included), and a large proportion of patients received total intravenous anesthesia."

Minor comment 1:

Some figures (especially multi-panel spline figures) are visually dense; clearer labeling or simplified legends would help readability.

Response to minor comment 1:

We believe that the figures should be kept as they are, but we have simplified their captions. Modifying the figures would result in a significant increase in their number.

Minor comment 2:

Minor language polishing is recommended to improve clarity and flow.

Response to minor comment 2:

We have reviewed the entire manuscript and made numerous changes that should address this recommendation.

Attachment Submitted filename: renamed_dd04d.docx

# Peer review: Decision Letter 1

**DOI:** 10.1371/journal.pone.0350048.r003

**Authors:** Vincenzo Lionetti

16 Mar 2026

PONE-D-25-54481R1

Association of intraoperative pulse pressure drop and minimum mean arterial pressure with postoperative length of stay: A stratified reanalysis of published data by age and sex

PLOS One

Dear Dr. Fischler,

Thank you for submitting your manuscript to PLOS ONE. After careful consideration, we feel that it has merit but does not fully meet PLOS ONE’s publication criteria as it currently stands. Therefore, we invite you to submit a revised version of the manuscript that addresses the points raised during the review process.

==============================

**ACADEMIC EDITOR:** all issues raised by expert reviewer are required.

==============================

Please submit your revised manuscript by Apr 30 2026 11:59PM. If you will need more time than this to complete your revisions, please reply to this message or contact the journal office at plosone@plos.org. When you're ready to submit your revision, log on to https://www.editorialmanager.com/pone/ and select the 'Submissions Needing Revision' folder to locate your manuscript file.

Please include the following items when submitting your revised manuscript:

- A letter that responds to each point raised by the academic editor and reviewer(s). You should upload this letter as a separate file labeled 'Response to Reviewers'.
- A marked-up copy of your manuscript that highlights changes made to the original version. You should upload this as a separate file labeled 'Revised Manuscript with Track Changes'.
- An unmarked version of your revised paper without tracked changes. You should upload this as a separate file labeled 'Manuscript'.

-->If you would like to make changes to your financial disclosure, please include your updated statement in your cover letter. Guidelines for resubmitting your figure files are available below the reviewer comments at the end of this letter.

If applicable, we recommend that you deposit your laboratory protocols in protocols.io to enhance the reproducibility of your results. Protocols.io assigns your protocol its own identifier (DOI) so that it can be cited independently in the future. For instructions see: https://journals.plos.org/plosone/s/submission-guidelines#loc-laboratory-protocols. Additionally, PLOS ONE offers an option for publishing peer-reviewed Lab Protocol articles, which describe protocols hosted on protocols.io. Read more information on sharing protocols at https://plos.org/protocols?utm_medium=editorial-email&utm_source=authorletters&utm_campaign=protocols.

We look forward to receiving your revised manuscript.

Kind regards,

Vincenzo Lionetti, M.D., PhD

Academic Editor

PLOS One

Journal Requirements:

1. If the reviewer comments include a recommendation to cite specific previously published works, please review and evaluate these publications to determine whether they are relevant and should be cited. There is no requirement to cite these works unless the editor has indicated otherwise.

2. Please review your reference list to ensure that it is complete and correct. If you have cited papers that have been retracted, please include the rationale for doing so in the manuscript text, or remove these references and replace them with relevant current references. Any changes to the reference list should be mentioned in the rebuttal letter that accompanies your revised manuscript. If you need to cite a retracted article, indicate the article’s retracted status in the References list and also include a citation and full reference for the retraction notice.

[Note: HTML markup is below. Please do not edit.]

Reviewers' comments:

Reviewer's Responses to Questions

**Comments to the Author**

1. If the authors have adequately addressed your comments raised in a previous round of review and you feel that this manuscript is now acceptable for publication, you may indicate that here to bypass the “Comments to the Author” section, enter your conflict of interest statement in the “Confidential to Editor” section, and submit your "Accept" recommendation.

Reviewer #1: (No Response)

Reviewer #2: (No Response)

**********

2. Is the manuscript technically sound, and do the data support the conclusions?

The manuscript must describe a technically sound piece of scientific research with data that supports the conclusions. Experiments must have been conducted rigorously, with appropriate controls, replication, and sample sizes. The conclusions must be drawn appropriately based on the data presented.

Reviewer #1: Yes

Reviewer #2: (No Response)

**********

3. Has the statistical analysis been performed appropriately and rigorously?

Reviewer #1: Yes

Reviewer #2: (No Response)

**********

4. Have the authors made all data underlying the findings in their manuscript fully available?

The PLOS Data policy requires authors to make all data underlying the findings described in their manuscript fully available without restriction, with rare exception (please refer to the Data Availability Statement in the manuscript PDF file). The data should be provided as part of the manuscript or its supporting information, or deposited to a public repository. For example, in addition to summary statistics, the data points behind means, medians and variance measures should be available. If there are restrictions on publicly sharing data—e.g. participant privacy or use of data from a third party—those must be specified.

Reviewer #1: Yes

Reviewer #2: (No Response)

**********

5. Is the manuscript presented in an intelligible fashion and written in standard English?

PLOS ONE does not copyedit accepted manuscripts, so the language in submitted articles must be clear, correct, and unambiguous. Any typographical or grammatical errors should be corrected at revision, so please note any specific errors here.

Reviewer #1: Yes

Reviewer #2: (No Response)

**********

6. Review Comments to the Author

Please use the space provided to explain your answers to the questions above. You may also include additional comments for the author, including concerns about dual publication, research ethics, or publication ethics. (Please upload your review as an attachment if it exceeds 20,000 characters)

Reviewer #1: Minor comment 1: Speculative interpretation of CumTimePP >61 mmHg

In the Discussion (lines 334–345), the authors hypothesize that CumTimePP >61 mmHg reflects hypertensive status based on indirect reasoning. While this is an interesting hypothesis, the language should be made more explicitly conditional throughout this paragraph. Phrases such as "we can assume" and "could represent the hypertensive population" would benefit from further hedging, and the authors should acknowledge that this interpretation remains unverified in the absence of direct adjustment for known hypertensive patients in the analysis.

Minor comment 2: Neurosurgical patient specificity in the Generalizability section

The Generalizability section notes the predominance of neurosurgical patients but does not discuss the implications. Neurosurgical patients represent the single largest subgroup (n=2,062) and are physiologically distinct with respect to cerebrovascular autoregulation and blood pressure targets. The authors should briefly acknowledge that intraoperative blood pressure thresholds in neurosurgical patients may differ from the general surgical population, which further limits generalizability beyond what is currently stated.

Minor comment 3: Typographical error in the text

In the Generalizability section, reference 39 is cited as "Flutier et al." whereas the reference list correctly identifies the first author as "Futier." This should be corrected for consistency.

Reviewer #2: (No Response)

**********

7. PLOS authors have the option to publish the peer review history of their article (what does this mean?). If published, this will include your full peer review and any attached files.

If you choose “no”, your identity will remain anonymous but your review may still be made public.

**Do you want your identity to be public for this peer review?** For information about this choice, including consent withdrawal, please see our Privacy Policy.

Reviewer #1: No

Reviewer #2: No

**********

[NOTE: If reviewer comments were submitted as an attachment file, they will be attached to this email and accessible via the submission site. Please log into your account, locate the manuscript record, and check for the action link "View Attachments". If this link does not appear, there are no attachment files.]

To ensure your figures meet our technical requirements, please review our figure guidelines: https://journals.plos.org/plosone/s/figures

You may also use PLOS’s free figure tool, NAAS, to help you prepare publication quality figures: https://journals.plos.org/plosone/s/figures#loc-tools-for-figure-preparation.

NAAS will assess whether your figures meet our technical requirements by comparing each figure against our figure specifications.

# Author comment: Author response to Decision Letter 2

**DOI:** 10.1371/journal.pone.0350048.r004

27 Apr 2026

We would like to thank the reviewer for its comments, which have led us to clarify or modify certain points. are shown in red and crossed out (suppression) and in blue (addition) in the marked file.

Minor comment 1: Speculative interpretation of CumTimePP >61 mmHg

In the Discussion (lines 334–345), the authors hypothesize that CumTimePP >61 mmHg reflects hypertensive status based on indirect reasoning. While this is an interesting hypothesis, the language should be made more explicitly conditional throughout this paragraph. Phrases such as "we can assume" and "could represent the hypertensive population" would benefit from further hedging, and the authors should acknowledge that this interpretation remains unverified in the absence of direct adjustment for known hypertensive patients in the analysis.

Response to minor comment 1: The reviewer is right to suggest that the text be written more cautiously. This has led to a revision of this paragraph: “In our study, a longer time spent above 61 mmHg of PP was associated with increased pPOLOS. The maximum risk of pPOLOS was reached quickly, mainly after the first two minute per hour of surgery. This pattern suggests a separation between patients with short versus prolonged exposure to elevated PP, which might correspond to non-hypertensive and hypertensive profiles, respectively. However, the hypothesis that CumTimePP > 61 mmHg reflects underlying hypertensive status remains speculative, as we did not directly adjust for or stratify by documented hypertension in this analysis, and should therefore be interpreted with caution and confirmed in future studies.”

Minor comment 2: Neurosurgical patient specificity in the Generalizability section

The Generalizability section notes the predominance of neurosurgical patients but does not discuss the implications. Neurosurgical patients represent the single largest subgroup (n=2,062) and are physiologically distinct with respect to cerebrovascular autoregulation and blood pressure targets. The authors should briefly acknowledge that intraoperative blood pressure thresholds in neurosurgical patients may differ from the general surgical population, which further limits generalizability beyond what is currently stated.

Response to minor comment 2: We would like to thank the reviewer for this very pertinent comment. It prompted us to add a sentence to this chapter that addresses it: “… However, neurosurgical patients, particularly those who have undergone surgery for an intracranial condition, may have impaired cerebral autoregulation, requiring blood pressure targets to be adjusted accordingly. Therefore, our findings cannot be generalized to this patient population….”

Minor comment 3: Typographical error in the text

In the Generalizability section, reference 39 is cited as "Flutier et al." whereas the reference list correctly identifies the first author as "Futier." This should be corrected for consistency.

Response to minor comment 3: We apologize for this typographical error, which has been corrected.

Attachment Submitted filename: renamed_e838f.docx

# Peer review: Decision Letter 2

**DOI:** 10.1371/journal.pone.0350048.r005

**Authors:** Vincenzo Lionetti

7 May 2026

Association of intraoperative pulse pressure drop and minimum mean arterial pressure with postoperative length of stay: A stratified reanalysis of published data by age and sex

PONE-D-25-54481R2

Dear Dr. Fischler,

We’re pleased to inform you that your manuscript has been judged scientifically suitable for publication and will be formally accepted for publication once it meets all outstanding technical requirements.

Within one week, you’ll receive an e-mail detailing the required amendments. When these have been addressed, you’ll receive a formal acceptance letter and your manuscript will be scheduled for publication.

An invoice will be generated when your article is formally accepted. Please note, if your institution has a publishing partnership with PLOS and your article meets the relevant criteria, all or part of your publication costs will be covered. Please make sure your user information is up-to-date by logging into Editorial Manager at Editorial Manager® and clicking the ‘Update My Information' link at the top of the page. For questions related to billing, please contact billing support.

If your institution or institutions have a press office, please notify them about your upcoming paper to help maximize its impact. If they’ll be preparing press materials, please inform our press team as soon as possible -- no later than 48 hours after receiving the formal acceptance. Your manuscript will remain under strict press embargo until 2 pm Eastern Time on the date of publication. For more information, please contact onepress@plos.org.

Kind regards,

Vincenzo Lionetti, M.D., PhD

Academic Editor

PLOS One

Additional Editor Comments (optional):

Reviewers' comments:

Reviewer's Responses to Questions

**Comments to the Author**

1. If the authors have adequately addressed your comments raised in a previous round of review and you feel that this manuscript is now acceptable for publication, you may indicate that here to bypass the “Comments to the Author” section, enter your conflict of interest statement in the “Confidential to Editor” section, and submit your "Accept" recommendation.

Reviewer #1: (No Response)

Reviewer #2: (No Response)

**********

2. Is the manuscript technically sound, and do the data support the conclusions?

The manuscript must describe a technically sound piece of scientific research with data that supports the conclusions. Experiments must have been conducted rigorously, with appropriate controls, replication, and sample sizes. The conclusions must be drawn appropriately based on the data presented.

Reviewer #1: Yes

Reviewer #2: Partly

**********

3. Has the statistical analysis been performed appropriately and rigorously?

Reviewer #1: Yes

Reviewer #2: I Don't Know

**********

4. Have the authors made all data underlying the findings in their manuscript fully available?

The PLOS Data policy requires authors to make all data underlying the findings described in their manuscript fully available without restriction, with rare exception (please refer to the Data Availability Statement in the manuscript PDF file). The data should be provided as part of the manuscript or its supporting information, or deposited to a public repository. For example, in addition to summary statistics, the data points behind means, medians and variance measures should be available. If there are restrictions on publicly sharing data—e.g. participant privacy or use of data from a third party—those must be specified.

Reviewer #1: Yes

Reviewer #2: Yes

**********

5. Is the manuscript presented in an intelligible fashion and written in standard English?

PLOS ONE does not copyedit accepted manuscripts, so the language in submitted articles must be clear, correct, and unambiguous. Any typographical or grammatical errors should be corrected at revision, so please note any specific errors here.

Reviewer #1: Yes

Reviewer #2: Yes

**********

6. Review Comments to the Author

Please use the space provided to explain your answers to the questions above. You may also include additional comments for the author, including concerns about dual publication, research ethics, or publication ethics. (Please upload your review as an attachment if it exceeds 20,000 characters)

Reviewer #1: The authors have satisfactorily addressed all three minor comments from the previous round.

The Discussion paragraph on CumTimePP > 61 mmHg has been appropriately revised with explicitly conditional language, and the authors now clearly acknowledge that the hypothesized link to hypertensive status remains speculative without direct adjustment for documented hypertension.

The Generalizability section now appropriately acknowledges that neurosurgical patients may have impaired cerebral autoregulation requiring different blood pressure targets, and that findings cannot be generalized to this population.

The typographical error ("Flutier" to "Futier") has been corrected.

The manuscript is suitable for acceptance in its current form.

Reviewer #2: (No Response)

**********

7. PLOS authors have the option to publish the peer review history of their article (what does this mean?). If published, this will include your full peer review and any attached files.

If you choose “no”, your identity will remain anonymous but your review may still be made public.

**Do you want your identity to be public for this peer review?** For information about this choice, including consent withdrawal, please see our Privacy Policy.

Reviewer #1: No

Reviewer #2: No

**********

# Editor report: Acceptance letter

**DOI:** 10.1371/journal.pone.0350048.r006

**Authors:** Vincenzo Lionetti

PONE-D-25-54481R2

PLOS One

Dear Dr. Fischler,

I'm pleased to inform you that your manuscript has been deemed suitable for publication in PLOS One. Congratulations! Your manuscript is now being handed over to our production team.

At this stage, our production department will prepare your paper for publication. This includes ensuring the following:

* All references, tables, and figures are properly cited

* All relevant supporting information is included in the manuscript submission,

* There are no issues that prevent the paper from being properly typeset

You will receive further instructions from the production team, including instructions on how to review your proof when it is ready. Please keep in mind that we are working through a large volume of accepted articles, so please give us a few days to review your paper and let you know the next and final steps.

Lastly, if your institution or institutions have a press office, please let them know about your upcoming paper now to help maximize its impact. If they'll be preparing press materials, please inform our press team within the next 48 hours. Your manuscript will remain under strict press embargo until 2 pm Eastern Time on the date of publication. For more information, please contact onepress@plos.org.

You will receive an invoice from PLOS for your publication fee after your manuscript has reached the completed accept phase. If you receive an email requesting payment before acceptance or for any other service, this may be a phishing scheme. Learn how to identify phishing emails and protect your accounts at https://explore.plos.org/phishing.

If we can help with anything else, please email us at customercare@plos.org.

Thank you for submitting your work to PLOS ONE and supporting open access.

Kind regards,

PLOS ONE Editorial Office Staff

on behalf of

Prof. Vincenzo Lionetti

Academic Editor

PLOS One
